# Preparing an orthopedic department for COVID-19: Lessons learned from reorganization and educational activities

**DOI:** 10.1080/17453674.2020.1817305

**Published:** 2020-09-10

**Authors:** Rune Dall Jensen, Magnus Bie, Anne Plønd Gundsø, Johannes Martin Schmid, Joachim Juelsgaard, Maria Louise Gamborg, Hanne Mainz, Jan Duedal Rölfing

**Affiliations:** a Corporate HR, MidtSim, Central Denmark Region, Aarhus; b Department of Clinical Medicine, Aarhus University, Aarhus; c Department of Orthopaedics, Aarhus University Hospital, Aarhus; d Department of Respiratory Disease and Allergy, Aarhus University Hospital; e Centre for Health Sciences Education, Aarhus University, Denmark

## Abstract

Background and purpose — The COVID-19 pandemic has disrupted healthcare services around the world. We (1) describe the organizational changes at a level 1 trauma center, (2) investigate how orthopedic healthcare professionals perceived the immense amount of information and educational activities, and (3) make recommendations on how an organization can prepare for disruptive situations such as the COVID-19 pandemic in the future.

Methods — We conducted a retrospective survey on the organizational restructuring of the orthopedic department and the learning outcomes of a needs-driven educational program. The educational activities were evaluated by a non-validated, 7-item questionnaire.

Results — The hospital established 5 COVID-19 clusters, which were planned to be activated in sequential order. The orthopedic ward comprised cluster 4, where orthopedic nursing staff were teamed up with internal medicine physicians, while the orthopedic team were redistributed to manage minor and major injuries in the emergency department (ED). The mean learning outcome of the educational activities was high–very high, i.e., 5.4 (SD 0.7; 7-point Likert scale). Consequently, the staff felt more confident to protect themselves and to treat COVID-19 patients.

Interpretation — Using core clinical competencies of the staff, i.e., redistribution of the orthopedic team to the ED, while ED physicians could use their competencies treating COVID-19 patients, may be applicable in other centers. In-situ simulation is an efficient tool to enhance non-technical and technical skills and to facilitate organizational learning in regard to complying with unforeseen changes.

The COVID-19 pandemic has caused an unprecedented destabilization of healthcare services on a global scale. In Denmark, the case fatality rate was 4.8% in June 2020 compared with the global case fatality rate of 5.4% (Dong et al. 2020).

As the COVID-19 pandemic spread throughout Europe, authorities started to reorganize inpatient care at hospitals to ensure the healthcare system would be able to treat the anticipated vast numbers of patients suffering from COVID-19. While research into medical treatment for COVID-19 is ongoing and the development of a vaccine still seems to be many months away, the pressure on the healthcare professionals continues to intensify in certain parts of the world. In spring 2020, the potentially overwhelming burden of illnesses stressed the Danish health system’s capacity and healthcare professionals. Hence, the widespread use of recommended personal protection equipment (PPE) in the care of all patients with respiratory symptoms had the highest priority (Adams and Walls 2020). In the orthopedic domain, Liang et al. (2020a, b) highlighted 3 main principles for surgeries during the pandemic; (1) determining the urgency of the surgery and the possibility of postponing it, (2) protecting the health of professionals and patients by providing PPE and rapid testing, and (3) being mindful of conserving healthcare resources (Liang et al. 2020a). Here, Hirschmann et al. (2020) pointed to the importance of being mindful of how to handle aerosol-generating procedures, which is especially prominent during orthopedic surgeries. Furthermore, interdisciplinary collaboration is argued to be essential for ensuring a high-quality information flow (Liang et al. 2020a).

At Aarhus University Hospital in Denmark, several learning modalities, e.g., in-situ simulation, e-learning, classroom teaching, etc. were used to effectively disseminate information and training to healthcare professionals to meet the demands of the COVID-19 disease.

The present study (1) describes the organizational changes of a level 1 trauma center in Denmark, (2) investigates how healthcare professionals in the Department of Orthopedics perceived the immense amount of information and training, and (3) makes recommendations on how an organization can prepare for disruptive situations such as the COVID-19 pandemic in the future.

## Methods

### Organizational restructuring

We conducted a retrospective survey on the organizational restructuring of the department and the learning outcomes of the needs-driven educational program at a level 1 trauma center (550–700 cases annually) and orthopedic department during COVID-19. The orthopedic department comprises 48 beds, 5 day beds, 300 employees, 3,000 elective orthopedic procedures, 3,200 day surgeries, 1,700 acute orthopedic procedures, and 34,000 outpatient visits annually. Data regarding the organizational restructuring of the hospital and the orthopedic department was obtained from the administration and the healthcare professionals involved.

### Questionnaire

**Figure 1.**
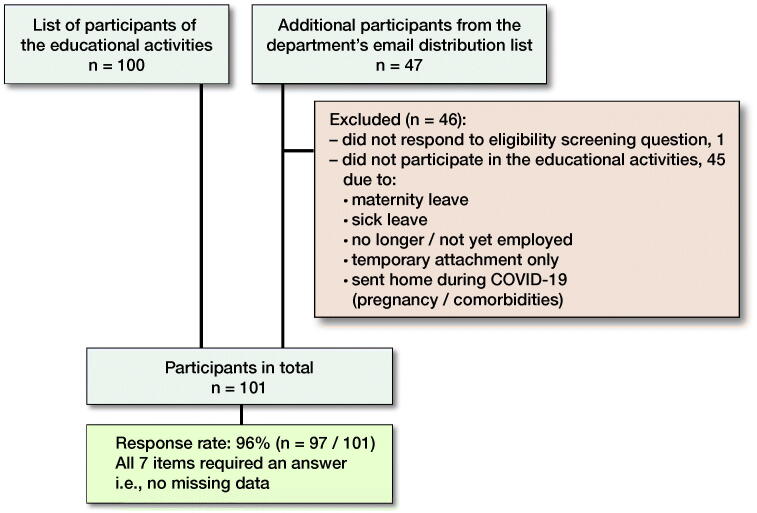
Questionnaire evaluating needs-driven educational activities: identification, eligibility of participants and response rate.

A non-validated, 7-item questionnaire (in Danish is available from the authors) regarding the healthcare professional’s perception of the educational activities and the learning outcome was developed. The questionnaire was distributed electronically to the participants (n = 101, Figure 1) via SurveyXact.dk (Rambøll Management Consulting, Aarhus, Denmark) allowing for tracking of individual participation/lack of participation but ensuring anonymous data analysis. Answer options for individual questions were displayed in random order for each individual participant. Participants had the opportunity to write comments at the end of the questionnaire. Data was collected from June 9, 2020 until June 18, 2020. Email reminders were sent twice. Furthermore, healthcare professionals reminded their colleagues to participate verbally and through social media.

### Statistics

The before/after items of the questionnaire were analyzed using 1-way repeated ANOVA with subsequent pairwise comparison of the 3 subitems. Descriptive statistics, i.e., mean (standard deviation [SD]) were applied for the other items of the questionnaire. A p-value of ≤ 0.05 was considered statistically significant.

### Ethics, funding, data sharing, and potential conflict of interests

Participation in the survey was voluntary and no payment was offered. Ethical approval of an anonymous data analysis was not required from the Danish Committee on Health Research Ethics (according to the Danish Ethical Committee Law § 14, stk. 2). The study was approved by the head of the orthopedic department and conducted in full accordance with ethical principles, including the World Medical Association Declaration of Helsinki. If a response rate > 90% was achieved, all 3 departments (orthopedic wards 1 + 2 and the outpatient clinic) were rewarded with candy for the entire staff. The original data, e.g., the Danish questionnaire and all participants’ responses, can be requested from the corresponding author. No external funding was received for this study. The authors declare that they have no conflicts of interest.

## Results

### Organizational changes at the hospital

Shocked by the devastating images of military lorries driving COVID-19 casualties to the crematorium in Northern Italy, social-distancing measures and lockdown of Danish society were carried out at an early stage. Aarhus University Hospital shut down all elective surgery and outpatient visits and started to reorganize the entire hospital for COVID-19 testing, triaging, resuscitation, and treatment. A stepwise approach was made in order to prepare inpatient departments to care for admitted COVID-19 patients in 5 newly established COVID-19 clusters. The first COVID-19 cluster was situated at the Department of Infectious Diseases, which started admitting COVID-19 patients at once, while the other departments were reorganized to prepare for this task. The next cluster should start admitting COVID-19 patients when 50% of the capacity of the previous cluster was in use. The COVID-19 clusters were headed by an infectious disease or respiratory medicine physician and the head of nursing from the respective departments.

### Organizational changes at the Department of Orthopedics

The Department of Orthopedics comprised cluster 4. Even at the height of the pandemic, only COVID-19 clusters 1 and 2 were activated, while clusters 3 and 4 used the time to prepare for handling the disease. Respiratory medicine physician and co-author JMS headed cluster 4 in co-leadership with the orthopedic head of nursing. COVID-19 cluster 4 was staffed by orthopedic nursing staff working in their usual workplace, but preparing and taking care of medically ill patients. Physicians in cluster 4 were recruited from the emergency department (ED) and the departments of endocrinology, geriatrics, and respiratory medicine.

### Orthopedic surgeons

Orthopedic surgeons (approximately 70, including residents) were redistributed to take care of minor and major injuries and trauma in the emergency department (ED) with a 2-day warning. This corresponded to 10 full-time employees. Meanwhile, ED physicians were deployed to staff COVID-19 testing, triaging, and COVID-19 clusters. This was a conscious choice by the administration, optimizing the use of core clinical competencies of the orthopedic team and ED physicians, who are more used to treating respiratory compromised patients suffering from multiple comorbidities. Consequently, this change caused minimal disruption and discomfort among orthopedic surgeons. Moreover, the usual orthopedic acute and subacute services provided by 2 consultants (24/7 and 8 am to 9 pm daily) and 1–2 residents (24/7) were still running 2 acute theaters daily instead of the usual 65 operating theatres on a weekly basis. The remaining orthopedic specialists were asked to work from home or take vacation. These changes took place from March 20, 2020 until May 4, 2020. Thereafter, the orthopedic surgeons as well as the ED staff returned to their regular jobs and orthopedic services gradually returned to normal conditions within 4–6 weeks.

During this period, the physical space of the ED was reused as a COVID-19 triaging area and the orthopedic outpatient clinic was therefore refurbished to house the ED, including a casting room and option to reposition fractures under fluoroscopy. However, standardized radiographs and CT scans were also available at the Department of Radiology. Orthopedic surgeons are usually in charge of trauma resuscitation at our level 1 trauma center receiving 550–700 trauma cases annually. Neither the organization of the trauma team nor the physical place were changed during COVID-19.

The educational activities “personal protection equipment (PPE)” and “hand hygiene” were also offered to orthopedic surgeons. However, almost none participated. Guidelines and availability of PPE when treating patients in the ED and when operating on patients with suspected or confirmed COVID-19 infection were often changing and resulted in uncertainty and discomfort among some orthopedic surgeons. Finally, no initiatives were made to acquire new surgical skills or prevent skill decay (Hedeman and Felländer-Tsai, 2020).

### Orthopedic nursing staff

Clinical competencies of the orthopedic nursing staff had to be refreshed and improved in order to be able to provide care to medical COVID-19 patients with multiple comorbidities instead of the regular orthopedic inpatients. Multiple needs-driven educational activities were initiated to enable the staff to protect themselves and their families from the infection and to provide care to COVID-19 patients.

### Educational activities preparing for COVID-19

A steering committee consisting of the head of the COVID-19 cluster and 4 administrative clinical nurses responsible for development, IT, and research ensured that the comprehensive flow of new relevant information from the Central Denmark Region to the hospital was reviewed and passed on to the staff through guidelines, newsletters, and daily morning meetings at the department. All questions and concerns from the staff were reviewed and addressed at staff meetings and on a question-and-answer poster. Based on the staff’s concerns and needs, several educational activities were planned, including the correct use of PPE, care for medical COVID-19 patients, etc. (Table 1). Educational activities were thus needs-driven, supplementing the competencies of the staff and addressing their concerns and suggestions. To ensure adaptability of the educational program, the participants conducted ad hoc assessment of the learning and relevance of the activities. These assessments were continuously evaluated by the committee, who discontinued inefficient initiatives and added new activities as requested. 2 specialty nurses were in charge of monitoring and ensuring attendance. Lists of participants in all activities were drawn up, and activities continued to be offered until the entire staff had the opportunity to attend. In order to prevent the spread of COVID-19 among participants, educational activities were offered multiple times per day, in small groups of 2–5 participants, in locations allowing for 1–2 m spacing between the participants.

**Table 1.** Needs-driven educational activities offered at the orthopedic department related to the COVID-19 pandemic response in order to prepare for COVID-19, ordered by mean (CI) recommendation on a 7-point Likert scale

Educational activity	Faculty, format, schedule, and objectives	Sequential order (needs-driven)	Duration	Participants (max. 101)	Recommend to colleagues mean (SD)
Full-scale in situ simulation team training	ABCDE approach to the patient with COVID-19, teamwork and communication, correct use of PPE	3	60 min	88	6.8 (0.5)
Use of personal protective equipment (PPE)	Donning and doffing procedures, types of PPE, precautions required regarding contact and aerosols, nursing and medical staff teamwork within the isolation room	1	Multiple courses mixed with simulation	92	6.4 (0.8)
Organizational structure of the COVID-19 department	E.g., distribution of doctors, examinations and tests, interdisciplinary cooperation	2	30 min	63	6.4 (0.8)
Arterial blood gas analysis	Arterial puncture analysis and using the machinery correctly, transport and timeframe for analysis; max. 2 participants/instruction	4	20 min	39	6.2 (1.2)
COVID-19 related deaths	Focus on the COVID-19 guidelines for deaths and the subsequent work practices	4	30 min	86	6.1 (0.9)
Lung physiotherapy	Patient positioning, mobilization, other aspects	1	30 min	85	6.1 (0.9)
Basic oxygen therapy and airway suctioning	Practical skills training	3	10 min	85	5.9 (1.1)
The elderly patient and medical issues	Educator launched discussion on considerations regarding the weakened elderly patient who has COVID-19	2	45 min	84	5.7 (1.1)
Fluid resuscitation and nutrition	Sufficient nutrition and correct use of feeding tubes, fluid restrictions related to COVID-19	2	30 min	81	5.6 (1.2)
Delirium—symptoms and causes	Interventions and confusion and assessment methods (CAM)	4	45 min	59	5.6 (1.3)
Hand hygiene	Additional focus to avoid contact transmission of COVID-19	1	E-learning and video	101	N/A
Following nursing staff at the Departments of Endocrinology and of Respiratory Medicine	Peer-to-peer work observation and teaching Discontinued due to inefficiency	1	7.4 hours	7	N/A

For chronological order please refer to the column sequential order (needs-driven).

Table 1 displays the educational activities, which were decisive initiatives to prepare the healthcare personnel in the orthopedic department for COVID-19.

### Questionnaire

97 of 101 participants in the educational activities answered the questionnaire (Figure 1).

The majority of educational activities received excellent feedback from the participants (Table 1).

The self-reported mean learning outcome of all attended educational activities was high–very high, i.e., 5.4 (CI 5.0–6.0) on a 7-point Likert scale with anchors 1 = extremely low, 2 = very low, 3 = low, 4 = neither/nor, 5 = high, 6 = very high, 7 = extremely high.

**Figure 2.**
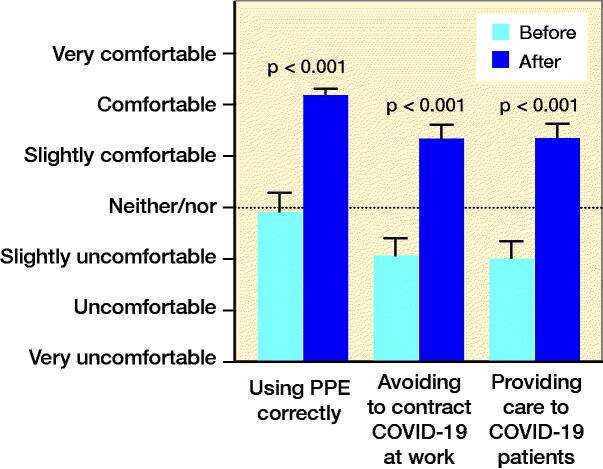
Mean results (CI) of the question: “Before/after participating in the educational activities, how comfortable did you feel about: (1) using PPE correctly?, (2) avoiding contracting COVID-19 at work?, (3) providing care to COVID-19 patients?

The participants felt more comfortable after participation in the educational activities in this unprecedented situation (Figure 2).

### Free text comments of the participants

The participants perceived that the COVID-19 situation resulted in ambivalent experiences for healthcare professionals. On one hand, participants perceived the organizational changes as positive (i.e., increase in interdisciplinary actions, decrease of bureaucracy, and a stronger sense of community). On the other hand, participants reported uncertainty, due to the severity of the disease combined with an overwhelming amount of information, as a participant wrote in the questionnaire:

“There was a lot of information to process. In addition, guidelines changed rapidly, making it very difficult to feel certain and up to date.”

Simulation training was perceived as key in preparing oneself and the department for the COVID-19 disease by de-mystifying the disease, and providing hands-on experiences with the patient group, effectively improving a sense of self-efficacy (Bandura 1994). Stress management especially was experienced as helpful in reducing potential stressors and increasing a sense of comfort in handling the COVID-19 patients, as also noted by participants in the survey stating that: “especially in-situ simulations were very fruitful”; “the introduction to treatment of COVID-19 patients provided some sense of security”; “After the [educational] activities, I felt so ready to do my part in fighting COVID-19 and treating patients”. 

## Discussion

In this retrospective survey, we describe the organizational changes for the entire orthopedic department and investigate how 101 healthcare professionals perceived different educational activities during the rapid global and national spread of COVID-19.

While healthcare workers accept an increased risk of infection as part of their chosen profession, they often exhibit concern regarding family transmission, especially involving family members who are elderly, immunocompromised, or have chronic medical conditions (Walton et al. 2020).

It is well known that stress and lack of commitment has a negative effect on healthcare professional quality of work. In surgery, Flin et al. (2006) showed that surgeons and nurses generally demonstrate positive attitudes to acts that are likely to enhance teamwork and safety in the operating theatre and surgical department (Flin et al. 2006). They found that the majority of nurses, surgeons, and trainees were more likely to make errors in tense and hostile situations. In our study, the participants reported that they experienced uncertainty in the rapid spread of the COVID-19 virus. Furthermore, they reported that the educational activities and in-situ simulation in particular made them feel more comfortable and helped to demystify the COVID-19 disease. This was further confirmed by the participants, who described a stronger sense of individual and interdisciplinary professional self-efficacy, less uncertainty, and lower stress levels after engaging in simulated training sessions.

These findings fit well with the revised model of stress by Palmer et al. (2003). Their model consists of 7 hazards for stress including employees’ demands and employees’ support and training. Demand is defined as the exposure to physical hazards and workload including insufficient personnel and complexity of work. COVID-19 is likely to add complexity of work in the orthopedic department. Hence, employees’ support and training seem essential in order to avoid stress and skill decay in a pandemic such as the COVID-19 disease (Kelc et al. 2020, Hedeman and Felländer-Tsai, 2020). Infrastructure is a key pillar supporting the fundamental aim of promoting improved standards of care and well-being for all patients, together with a good experience of the healthcare system (Luxon 2015). The data from the present study and data from a related study (focus-group interviews evaluating the efficacy of in-situ simulation during COVID-19, unpublished data) showed that learning occurred at an organizational level as well as an individual level and that an adaptable infrastructure at the hospital seems essential in order to treat the potentially increasing number of COVID-19 patients. A variety of different learning activities helped participants to acquire knowledge of different aspects of receiving and caring for COVID-19 patients. In-situ simulation was described as an efficient tool for learning how to deal with stressful situations, enhancing effective communication, and acquisition of specific technical skills. Furthermore, the instructor was mentioned as a key factor by being engaged in the scenario, ensuring fidelity, and securing a safety net by debriefing the simulation.

Another general insight from the participants was the impact and importance of interdisciplinary simulation training sessions. It highlighted each healthcare professional’s value and role when handling these patients, but also in general. Furthermore, interdisciplinary learning was facilitated in the simulated settings, in regard to specific workflows and knowledge-sharing, which again led to an increased sense of comfort in teamwork and trust in the handling of COVID-19.

Simulation has a huge potential to help in managing the global COVID-19 crisis in 2020 and in potentially similar future pandemics (Lababidi et al. 2020). Simulation can rapidly facilitate hospital preparation and education of large numbers of healthcare professionals and has proven its value in many settings (Dieckmann et al. 2020). It can be utilized to scale-up workforce capacity through experiential learning. Simulation and simulation facilitators can also contribute to the optimization of work structures and processes (Brydges et al. 2020).

### Limitations

This study has 3 main limitations. First, a non-validated retrospective questionnaire was utilized exploring healthcare professionals’ experience of a variety of educational activities during the COVID-19 pandemic. Second, recall bias is likely to occur in retrospective surveys, but, due to the rapid development of COVID-19 and immediate introduction of the first learning activities (Table 1), prospective data collection was not feasible (Althubaiti 2016). Third, 1 item in the questionnaire assessed the learning outcome of the activities attended. However, subjective assessment of the learning outcome of an educational intervention is not as accurate as an objective assessment, i.e., an examination or practical test of what was learned (Lababidi et al. 2020). Nonetheless, the educational activities built confidence and may thus have decreased stress and other mental health problems, which healthcare professionals are prone to during the current health crisis (Walton et al. 2020).

Recommendations and lessons learned from reorganizing a level 1 trauma center and orthopedic department to prepare and handle COVID-19 are summarized in Table 2. In particular, in-situ simulation received excellent evaluations and may prove useful in preparing staff for COVID-19 and other disruptive events challenging the core clinical competencies. Many healthcare professionals experienced uncertainty due to the rapid development of the COVID-19 disease.

**Table 2.** General recommendations for orthopedic departments on how to use competencies and establish needs-driven educational program in order to prepare for unprecedented situations

Prepare • Establish a steering committee to facilitate and support continuous learning activities based on relevant guidelines and the staff’s need and feedback. • Prepare 2–3 educational activities with relevance to protecting employees, i.e., in-situ simulation focusing on use of personal protection equipment and hygiene. Facilitate • Conduct interdisciplinary in-situ simulation. • Adapt simulation scenarios to the needs of the given department/staff. • Conduct ad hoc assessments of educational program to promote needs-driven initiatives. Organize • Establish knowledge-sharing across departments/clusters. • Use competencies and resources wisely. Redistributing orthopedic surgeons to manage minor and major orthopedic injuries in the ED is a minor disruption compared with taking care of medically ill patients suffering from COVID-19. This use of core clinical competencies may be applicable in a large variety of hospitals in Scandinavia and around the world.
